# Main group carbonyl complexes

**DOI:** 10.1038/s42004-020-00423-9

**Published:** 2020-11-23

**Authors:** Shiori Fujimori, Shigeyoshi Inoue

**Affiliations:** Department of Chemistry, WACKER-Institute of Silicon Chemistry and Catalysis Research Center, Technische Universität München, Lichtenbergstraße 4, 85748 Garching bei München, Germany

**Keywords:** Chemical bonding, Coordination chemistry

## Abstract

The chemistry of carbon monoxide (CO) as a ligand has evolved significantly and transition-metal carbonyl complexes have been widely used as catalysts in many important catalytic processes. Here the authors comment on the recent progress of main-group element carbonyl complexes along with their future prospects.

Main-group elements belonging to the *s*- and *p*-blocks of the periodic table contain some of the most abundant elements in the Earth’s crust (e.g., silicon and aluminium) and their compounds are manufactured as commercially valuable chemicals. Main-group chemistry has been studied significantly and various main-group compounds in low-valent oxidation states bearing a high-energy highest occupied molecular orbital and an energetically accessible lowest unoccupied molecular orbital are known to mimic the reactivity of transition-metal (TM) complexes such as the activation of small molecules and catalytic reactions.

Recently, the chemistry of main-group carbonyl complexes has garnered increased attention. Carbon monoxide (CO), a ubiquitous atmospheric trace gas produced by natural and anthropogenic sources, is a versatile feedstock for chemical or material production and is widely utilized in both academia and industry. In organometallic chemistry, CO is an important ligand (carbonyl) and plays a key role in various catalytic processes. TMs are known to react with CO to form TM carbonyl complexes with various types of TM–CO bonding motifs, e.g., terminal, bridging, and isocarbonyl. The most common TM carbonyl complexes contain terminal CO bonds, which consists of σ-donation from CO lone pair to an empty orbital on the TM and π-backdonation from a filled *d* orbital of the TM to an empty π*-orbital on CO. These complexes are used as important reagents in various industrial applications such as hydroformylation, the Cativa process, the Fischer–Tropsch process, and the Pauson–Khand reaction^1^.

## Main-group carbonyl complexes

**Fig. 1:**
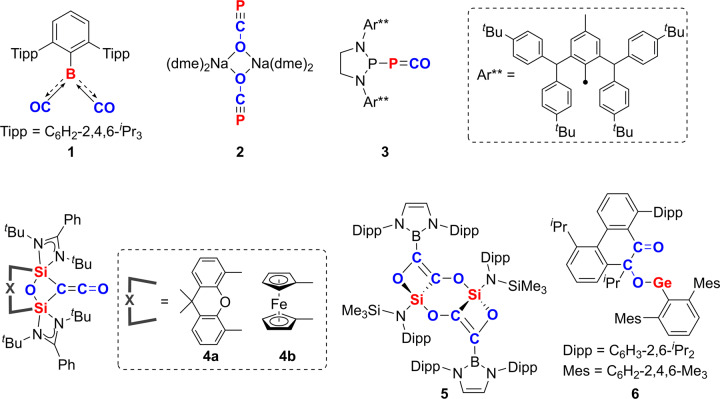
Selected examples of main-group element compounds formed in the reactions with CO. Boron dicarbonyl complex (**1**)^5^, sodium phosphacyanate (**2**)^7^, phosphino-phosphaketene (**3**)^8^, and reductive homologation products (**4**–**6**)^11–13^.

Although the formation of carbonyl complexes by the simple reaction with CO is very rare for main-group elements, due to the lack of suitable π-back-bonding orbitals, some milestone advances in main-group carbonyl chemistry have been recently reported. For the *s*-block elements, only alkaline Earth metal complexes M(CO)_8_ (M = Ca, Sr, or Ba), which are isolated in a low-temperature neon matrix, have been reported by the groups of Zhou, Frenking and colleagues^2,3^. On the other hand, several examples of isolable *p*-block carbonyl complexes at ambient temperature have been reported. It is known that Lewis acidic boranes such as BH_3_, HB(C_6_F_5_)_2_, and perfluoroalkylboranes react with CO to form the corresponding boron mono-carbonyl complexes, which have a weak CO–B σ-bonding^4^. Some of the boron mono-carbonyl complexes are known to undergo nucleophilic attack by bases such as NH_3_ at the carbon atom of the carbonyl. Furthermore, a boron multi-carbonyl complex, the dicarbonyl compound TpB(CO)_2_ (Tp = 2,6-[2,4,6-^*i*^Pr_3_-C_6_H_2_]_2_-C_6_H_3_) (**1**), was isolated by Braunschweig et al.^5^ (Fig. 1). Compound **1** photolytically liberates CO, similar to TM carbonyl complexes^6^. Besides boron carbonyl complexes, several reactions of phosphorus compounds with CO have been studied. Grützmacher et al.^7^ have isolated sodium phosphacyanate, [Na(O–C≡P)(dme)_2_]_2_ (**2**), by the reaction of sodium dihydrogen phosphide NaPH_2_ and CO. In addition, the Bertrand and colleagues^8^ reported the synthesis of the phosphino-phosphaketene (R_2_P–P=C=O) (**3**), which can proceed via a ligand exchange reaction at the main-group element center, one of the most prototypical reaction of TM carbonyl complexes.

With regard to Group 14 elements, some studies on the reactivity of stable carbenes (:CR_2_) with CO have been demonstrated. For instance, the Bertrand and colleagues^9^ revealed that the reaction of stable alkyl(amido)carbenes with CO afforded the isolable amido ketenes [C(N)C=C=O] at ambient temperature. Thus, the reactions of carbenes (:CR_2_) with CO results in the formal oxidation of carbenes to ketenes (R_2_C=C=O) rather than the formation of Lewis adducts (R_2_C ← CO). In general, reactivity of low-valent main-group compounds towards CO is dominated by reductive homologation reactions, even without cooperative effects by alkali metals or TMs^10^. In fact, CO homologation was observed in the reaction of tetrylene (:ER_2_, E = Si, Ge), the heavier analogs of carbenes (:CR_2_); therefore, the isolation of heavier Group 14 element carbonyl complexes is challenging. Driess and colleagues^11^ reported the reaction of bis-silylenes with CO, which resulted in the selective deoxygenative homocoupling of CO to form compounds **4a** and **4b**. In addition, the reaction of an acyclic amido(boryl)silylene with CO was demonstrated by Aldridge and colleagues^12^. In this reaction, reductive coupling of CO, followed by insertion reaction into the Si–B bond was observed to yield compound **5**^12^. Furthermore, Power and colleagues^13^ revealed that stable germylenes (:GeR_2_) react with CO to obtain compound **6** via C–C bond cleavage and formation.

**Fig. 2:**
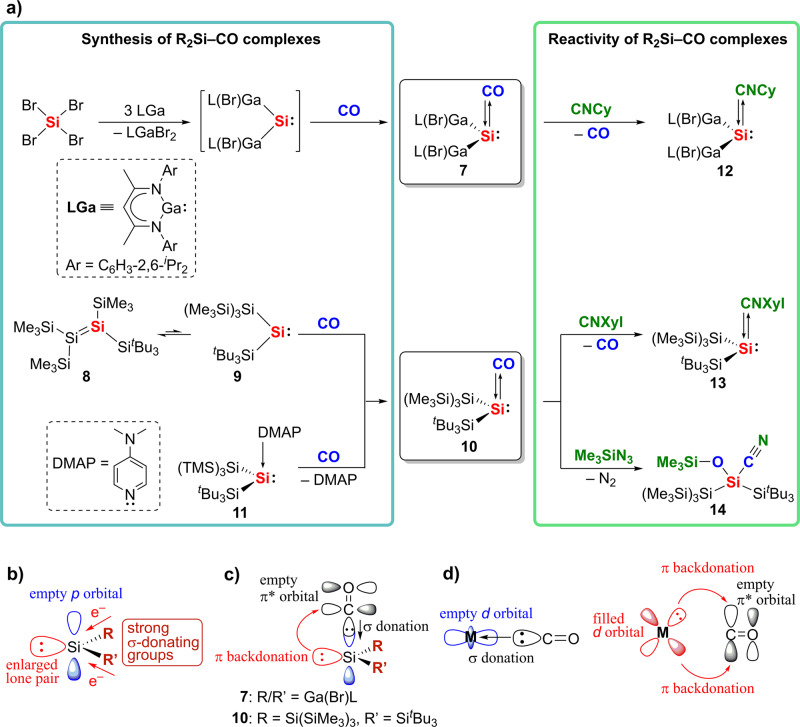
Silicon carbonyl complexes. Synthesis of silicon carbonyl complexes (**7** and **10**) and their reactivity (**a**). Important molecular orbitals in silylenes bearing electropositive groups (**b**) and the frontier orbital interactions in silicon carbonyl complexes (**c**). Frontier orbital interactions in transition-metal (M) carbonyl complexes (**d**). CNCy, cyclohexyl isocyanide; CNXyl, 2,6-dimethylphenyl isocyanide.

Very recently, the first synthesis and isolation of a room-temperature stable carbonyl complex of heavier Group 14 element has been achieved by tuning the substituents on the central element. The groups of Schreiner, Schulz and colleagues^14^ prepared silicon carbonyl complex [L(Br)Ga]_2_Si–CO (L = HC[C(Me)N(2,6-^*i*^Pr_2_-C_6_H_3_)]_2_) (**7**) bearing a gallium-based ligand framework, as orange crystals in 75% yield, by the reaction of GaL with SiBr_4_ under a CO atmosphere (Fig. 2a). Shortly after, our group succeeded in the synthesis and isolation of the silyl-substituted silicon carbonyl complex, [(Me_3_Si)_3_Si](^*t*^Bu_3_Si)Si–CO (**10**), which is stable at ambient temperature^15^. Complex **10** was obtained as purple crystals in 90% yield on exposure of an equilibrium mixture of tetrasilyldisilene (**8**) and bis(silyl)silylene (**9**) to CO. Alternatively, the reaction of 4-*N,N*-dimethylaminopyridine-stabilized bis(silyl)silylene (**11**) with CO also afforded **10**. Furthermore, complex **10** can photolytically decarbonylate to generate an equilibrium mixture of **8** and **9**. Both complexes **7** and **10** show remarkably stability both in the solid state (*T*_d_ = 176–177 °C for **7**, 76–77 °C for **10**) and in solution. Our group investigated that the silicon carbonyl complex (^*t*^Bu_3_Si)_2_Si–CO could also be obtained by the reaction of (^*t*^Bu_3_Si)_2_SiBr_2_ and potassium graphite (KC_8_) in a CO atmosphere, whereas acyclic imino(silyl)- and imino(siloxy)silylenes showed no reaction towards CO.

Further insight regarding the chemical bonding in the silicon carbonyl complexes was gained by nuclear magnetic resonance (NMR) and infrared (IR) spectroscopy. Complexes **7** and **10** show a downfield signal for CO in the ^13^C NMR spectrum and an enormous upfield signal for the Si–CO moiety in the ^29^Si NMR spectrum; notably, a red shift for the CO band was observed in IR spectroscopy. These results indicate a significantly stronger donor–acceptor interaction on the Si–CO moiety. Both complexes **7** and **10** bear electropositive groups (gallyl group for **7**, silyl groups for **10**), which have strong σ-donating properties (Fig. 2b). The polarization of the Si–CO moiety and the electron density at the silicon center are increased by the effect of these electropositive ligands, which induces π-backdonation from the silylene centers to the CO ligands. Their analytical data together with theoretical studies revealed that complexes **7** and **10** exhibit strong donor–acceptor CO–Si bonding, with CO → Si σ donation and Si→CO π-backdonation, which is evocative of TM carbonyl complexes (Fig. 2c, d).

The successful isolation of silicon carbonyl complexes has also led to an investigation of their reactivity. Complexes **7** and **10** exhibit a ligand exchange reaction, which is archetypal for TM carbonyl complexes. Silylene isocyanide complexes [L(Br)Ga]_2_Si–CNCy (**12**) and [(Me_3_Si)_3_Si](^*t*^Bu_3_Si)Si–CNXyl (**13**) were obtained by the reactions of **7** and **10** with isocyanides (R–CN), respectively. The stronger σ-donation of the isocyanides, compared to that of CO, should be the driving force. In addition, complex **10** shows a functionalization reaction of the CO moiety, which is also a prototypical reaction for TM carbonyl complexes. The reaction of **10** with trimethylsilyl azide (Me_3_SiN_3_) afforded the silyl cyanide [(Me_3_Si)_3_Si](^*t*^Bu_3_Si)Si(OSiMe_3_)(CN) (**14**). Thus, complexes **7** and **10** show reactivity as TM mimics.

## Outlook

Thanks to the enormous recent progresses in main-group chemistry, several main-group element carbonyl complexes including the silicon analogs were isolated as stable compounds and their structural motif and chemical behavior has come to light. However, the isolable carbonyl complexes of many *p*-block elements such as heavier Group 13 elements including aluminium, the third most abundant element, are still missing. The heavier Group 14 analogs, germanium and tin carbonyl complexes R_2_E–CO (E = Ge, Sn), have also not been reported but could be candidates for application in catalytic cycles. Contrary to the case of silicon, the divalent states in germanium and tin species are relatively stable, which may induce a migratory insertion of the coordinated CO in the germanium/tin carbonyl complexes. These complexes may provide viable catalysts instead of TM complexes for important catalytic reactions in industrial applications. To isolate these carbonyl complexes as stable compounds, appropriate molecular design is required. Given the success in the synthesis of the silicon carbonyl complexes [L(Br)Ga]_2_Si–CO **7** and [(Me_3_Si)_3_Si](^*t*^Bu_3_Si)Si–CO **10**, the introduction of bulky electropositive groups such as silyl groups to the Ge/Sn element center is effective to stabilize the corresponding carbonyl complexes. The bond energy of E–Si(silyl group) (E = Ge, Sn) bond in the potential complexes (R_3_Si)_2_E–CO is weaker than when E = Si (Si–Si: 327 kJ mol^−1^, Si–Ge: 301 kJ mol^−1^), which also may be a driving force for a migratory insertion. Although TM complexes have been widely used as highly efficient catalysts for various important reactions, the development of TM free and eco-friendly systems have been underexplored. We believe that the ability of the main-group element carbonyl complexes may enable the widespread development of main-group element catalysis as alternatives to TMs and for new catalytic processes in the future.
